# Comparison of tibial anatomical-mechanical axis angles and patellar positions between tibial plateau levelling osteotomy (TPLO) and modified cranial closing wedge osteotomy (AMA-based CCWO) for the treatment of cranial cruciate ligament disease in large dogs with tibial plateau slopes greater than 30° and clinically normal Labradors retrievers

**DOI:** 10.1186/s12917-021-03094-3

**Published:** 2021-12-03

**Authors:** Laurent Guénégo, Aldo Vezzoni, Luca Vezzoni

**Affiliations:** 1 Clinique Vétérinaire du Vernet, 366 avenue de Labarthe, 31810 Le Vernet, France; 2 Clinica Veterinaria Vezzoni, Via delle vigne, 190, 26100, Cremona, Italy

**Keywords:** Cranial cruciate ligament, patella, Canine, Tibial anatomic axis, Tibial mechanical axis, AMA angle, TPA, TPLO, CCWO

## Abstract

**Background:**

The objective of this study was to evaluate tibial anatomical-mechanical axis angles (AMA-angles) and proximodistal and craniocaudal patellar positions following tibial plateau levelling osteotomy (TPLO) and AMA-based modified cranial closing wedge osteotomy (CCWO) in large dogs with tibial plateau angle (TPA) > 30°, to compare these postoperative positions with those of a control group of healthy normal dogs, and to assess which procedure yields postoperative morphology of the tibiae and stifles that is most consistent with that of the unaffected group. This study also investigated whether the occurrence of patellar ligament thickening (PLT), which is commonly observed 2 months postoperatively after TPLO, is associated with misplacement of the osteotomy. A total of 120 dogs weighing more than 20 kg, 40 of which were control animals, were enrolled in this retrospective study. Stifles were radiographically evaluated preoperatively and postoperatively on the side with CCLR and on the healthy contralateral side and compared with clinically normal stifles. PLT was reassessed after 2 months.

**Results:**

Significant decreases in median patellar height ratio were found after both procedures (TPLO 0.24 (0.05–0.8); CCWO 0.22 (0.05–0.4)). The postoperative craniocaudal patellar position and the median AMA angle differed significantly among the groups (*P =* 0.000) (TPLO 87.5% caudal to the AA and 3.12° (0.76–6.98°); CCWO 100% cranial to the AA and 0° (− 1.34–0.65°); control group 5% caudal to the AA and 0.99° (0–3.39°)).

At 8 weeks, PLT grade differed significantly in the two operated groups (*P =* 0.000) (TPLO 40% 0–2, 20% 2–4, 40% > 4; CCWO 98.8% 0).

**Conclusions:**

TPLO and AMA-based CCWO are associated with significant decreases in patellar height; however, the PLT results 2 months postoperatively differed between the two groups; the decrease in patellar height and PLT were independent of osteotomy position in the TPLO group. Compared to TPLO, CCWO results in reduced postoperative AMA angles and craniocaudal patellar positions that more closely resemble those of unaffected dogs, suggesting that the CCWO procedure allows us to better correct the caudal bowing of the proximal tibia that is often associated with deficient stifles in large dogs with TPA > 30°.

## Background

Controversy exists regarding the best surgical treatment for cranial cruciate ligament rupture (CCLR) in dogs. These treatments include extracapsular stabilization and procedures in which osteotomies are used to modify the biomechanics of the stifle [1]. Tibial plateau levelling osteotomy (TPLO) [1–3] and cranial closing wedge osteotomy (CCWO) are common osteotomy procedures [4–10] that alter the tibial plateau slope (TPS) [11]. Although numerous studies in which the clinical outcomes and complication rates of these procedures were compared have failed to demonstrate the superiority of any one technique [4, 6], one major disadvantage of CCWO that has been cited is that it produces a change in proximodistal patellar position resulting from a more craniodistal position of the tibial crest, leading to stifle and hock hyperextension to compensate for patella baja (PB) [11–13]. However, the majority of orthopaedic surgeons consider the occurrence of patellar height decrease and PB following TPLO to be avoidable [4].

Postoperative PB can be divided into true PB (TPB) [14] and pseudo-PB (PPB) [15]. Postsurgical PPB can be secondary to a change in proximal tibial anatomy caused by surgical TPS alteration in the presence of a normal patellar tendon, as seen with CCWO, and has been assessed in a previous study using the Guenego-Verwaerde Index (GVI) [16]. TPB has been evaluated using the Insall-Salvati Index (ISI) [14, 17]; it can result from a postoperative decrease in the distance between the distal pole of the patella and the tibial crest and could be exacerbated by misplacement of the radial osteotomy, as reported by Kowaleski et al. [18] in dogs treated with TPLO.

This alteration of the distance between the patella and the proximal tibial segment may have clinical consequences, as patellar ligament thickening (PLT) is often observed 2 months after the TPLO procedure [19, 20]; it has not been reported in previous studies to occur after CCWO [7, 16].

The craniocaudal position of the patella relative to the tibial distal anatomical axis (AA) is correlated with the angle between the AA and the mechanical axis (MA), called the AMA angle; this angle quantifies the craniocaudal angulation of the proximal tibia [21, 22]. The AMA angle has recently been recognized as a predisposing factor in the development of CCLR. Indeed, a recent study in which dogs at low risk of developing CCLR were compared with a consecutive series of dogs with surgically confirmed CCLR showed that an AMA angle higher than 1.9° had a sensitivity of 0.94 and a specificity of 0.97 in predicting CCLR [22], and another study in which Labrador retrievers and Golden retrievers with and without CCLR were compared found that an AMA angle equal to or greater than 2.4° was predictive of CCLR with a sensitivity of 0.95 and a specificity of 0.95 [21].

Thus, it has been suggested that the AMA angle should be taken into consideration when a TPS alteration procedure must be chosen, especially since the AMA angle and TPA are strongly correlated with TPA > 30° [21, 22].

The purpose of this retrospective radiographic descriptive study was to quantify changes in AMA angle and patellar position following TPLO and a modified CCWO, AMA-based CCWO [7] in a subset of dogs with TPA > 30° and to compare these postoperative positions with those observed in a control group of healthy normal dogs to determine which procedure yields postoperative morphology of the tibiae and stifles that is most consistent with that of the unaffected group.

We hypothesized that patellar height would decrease following TPLO and AMA-based CCWO, even when these procedures were performed by surgeons experienced in the relevant techniques, and that no postoperative PB would occur but that the two procedures would result in different changes in the patellar ligament at two-month follow-up, with patellar height decrease and PLT occurring regardless of the osteotomy position in the TPLO group. We also hypothesized that the craniocaudal position of the patella following TPLO would differ from that of a normal stifle, while after AMA-based CCWO, a patellar position more consistent with that of the dogs in the control group was expected.

## Results

### Dogs

The demographics of 120 dogs were available for this study (Table 1). As expected based on the inclusion criteria, the dogs in the control group were older than the dogs that were treated for CCLR, but the dogs in these two groups did not differ significantly in body weight (*P =* 0.005) or sex (*P =* 0.071). There was no significant difference between the dogs that received TPLO and those that received AMA-based CCWO in mean body weight (*P =* 0.063), the incidence of side effects (*P =* 0.653) or sex (*P =* 0.198).

**Table 1:** Characteristics of the study population

Median: min – max	Control group	TPLO group	CCWO group
Number of dogs	40	40	40
Age (years)	12.1 (11.2–16)	3.4 (0.8–13)	3.7 (1.2–8.6) *
Weight (kg)	37 (27–56)	31.5 (20.6–64.8)	36 (20–62)
Sex (M(neut.)/F(spayed))	21(3)/19	14(1)/26(25)	17(1)/23(23)
Breeds included:			
Labrador Retriever	40	11	3
Golden Retriever		3	4
Rottweiler		0	4
Am. Staff Terrier		2	3
Boxer		1	4
Cane Corso		1	4
Bernese Mount Dog		3	2
Bulldog		4	3
Australian Shepherd		1	3
Mixed, other breeds		14	10

* *P* < 0.05 using a Mann-Whitney nonparametric comparison

### Tibial measurements

Preoperative TPA did not differ significantly between the TPLO and AMA-based CCWO groups (*P =* 0.082) (TPLO 31° (30–37°); AMA-based CCWO 31.1° (30–49.6°)), but the preoperative TPAs of these two groups differed from that of the control group (26.3° (20.4–30.8°)) (*P =* 0.000).

Postoperative TPA differed significantly in the TPLO and AMA-based CCWO groups (*P =* 0.000) (TPLO 5° (2–8°); AMA-based CCWO 10° (6.2–15°)). However, the difference between the desired target TPA (TPAd) and the actual postoperative TPA (TPApo) was not significantly different between the two groups (*P =* 0.002) (TPLO 0° (− 3–3°); AMA-based-CCWO -0.1° (− 3.4–5°)).

The preoperative AMA angles of the TPLO and AMA-based CCWO groups were not significantly different (*P =* 0.285) (TPLO 3.54° (0.77–6.78°); AMA-based CCWO 3.45° (1.92–4.8°)), but the preoperative AMA angle in both of those groups differed significantly from that of the control group (*P =* 0.000) (0.99° (0–3.39°)).

In the TPLO and CCWO groups, 77.5% of the dogs had AMA angles > 3°.

The postoperative AMA angles of the two groups differed significantly (*P =* 0.000) (TPLO 3.26° (0.76–6.98°); AMA-based CCWO 0° (− 1.5–0.64°)), but that of the AMA-based CCWO group did not differ significantly from the AMA angle of the control group (*P =* 0.000) (0.99° (0–3.39°)).

In the AMA-based CCWO group, the cranial wedge osteotomy angle (CWO angle) was 20° (19–30°), the length of the cortical bone opposite the wedge was 10 mm (6–13.8 mm), and the decrease in tibial length was 3.5 mm (1–7.6 mm).

The tibial long axis shift (TLAS) differed between the two groups (*P =* 0.000). In the TPLO group, TLAS occurred in 100% of the cases and had a mean value of − 0.39° (− 1.63–1.4°), while in the AMA-based CCWO group, TLAS occurred in 7/40 dogs (17.5%), with a mean value of 0° (− 1.5–0.64°).

### Evaluation of patellar positions

#### Proximodistal patellar positions

The decrease in ISI after TPLO (ISIcls – ISIpo) was 0.24 (0.05–0.8), reflecting a decrease in the distance between the distal pole of the patella and the tibial crest (ISIcls 2.27 (1.91–2.64) and ISIpo 1.96 (1.62–2.39)).

The decrease in GVI after AMA-based CCWO (GVIcls-GVIpo) was 0.22 (0.05–0.4), reflecting a decrease in the distance between the distal pole of the patella and the tibial plateau (GVI-cls 1.59 (1.38–1.85) and GVIpo 1.35 (1.15–1.76)).

Thirteen (32.5%) dogs had ISIpo values between 1.6 and 1.9, which was considered low height in the TPLO group, and 7 dogs (17.5%) had GVIpo values between 0.9 and 1.25, which was considered low height in the AMA-based CCWO group. In both of these groups, the study population was distributed into either the normal height or low height classes, and there were no cases of PB (no TPB in the TPLO group, no PPB in the AMA-based CCWO group).

#### Craniocaudal patellar position

Postoperatively, in the TPLO group, 82.5% of the patellae were recorded as caudal to the AA, while in the AMA-based CCWO group 100% of the patellae were cranial to the AA; in the control group, 5% of the patellae were recorded as caudal to the AA.

### Patellar tendon thickening

At the two-month follow-up, there was a significant difference between the ISI of the control group and that of the TPLO group (*P =* 0.000) (control group ISI 2.32° (2.02–2.6°); TPLO group ISI 1.96 (1.32–2.49)).

PLT grade differed significantly between the two groups (*P =* 0.000) (TPLO group, PLT at the level of the patella (PLTp) grade 1 (0–4), PLT at the level of the tibial crest (PLTtc) grade 3 (0–5); AMA-based-CCWO group, PLTp grade 0 (0–2), PLTtc grade 0 (0–1)). In the TPLO group, PLTtc grade 0–2 was recorded for 16 dogs (40%), grade 2–4 was recorded for 8 dogs (20%), and grade > 4 was recorded for 16 dogs (40%).

There was no significant difference among the grade 0–2, 2–4 and > 4 groups in age (*P =* 0.495), weight (*P =* 0.148), TPA (*P =* 0.511), AMA-angle (*P =* 0.784), ISIpo (*P =* 0.683) or TLAS (*P =* 0.254), but there was a significant difference in the magnitude of the patellar height decrease in the grade > 4 group (*P =* 0.017) (Table 2). Compared with other grades, grade > 4 had a higher TLAS of 0.62° (0.1–1.1°) and a greater patellar height decrease of 0.42 (0.19–0.6).

**Table 2:** Median (range) characteristics of the TPLO group, tibial measurements, patellar height decreases and PLTc grades

PLTtc grade	0–2	2–4	> 4
Number of dogs (%)	16 (40)	8 (20)	16 (40)
Age (years)	3.6 (0.8–8)	3 (1.1–8)	4.5 (1.5–13)
Weight (kg)	35 (20.6–64.8)	32 (21–42)	29.4 (21–34)
Sex (M(neut.)/F(spayed))	8/10 (9)	5 (1)/9 (9)	1/7 (7)
TPA (°)	31 (30–37)	31 (30–36)	31 (30–34)
AMA angle (°)	3.6 (1.86–6.16)	3.84 (2.05–5.58)	3.44 (0.77–6.78)
TLAS	−0.28 (−1.63–0.4)	− 0.3 (− 0.81–1.4)	−0.62 (− 0.01–1.1)
ISIpo	1.94 (1.7–2.33)	2 (1.73–2.14)	1.94 (1.62–2.39)
ISIcls-ISIpo	0.21 (0.05–0.47)	0.29 (0.1–0.49)	0.42 (0.19–0.6) *

*PLTtc* Patellar ligament thickening at the level of the tibial crest, *TPA* Tibial plateau angle, *AMA angle* Angle between the AA and the MA, *TLAS* Tibial long axis shift, *ISIpo* Insall-Salvati index postoperatively, *ISIcls* Insall-Salvati index contralateral side. ISIcls–ISIpo represents the decrease in the distance between the distal pole of the patella and the tibial crest

* *P* < 0.05 using a Mann-Whitney nonparametric comparison

In the TPLO group, five osteotomy positions were recorded: E for 16 dogs (40%), D for 12 dogs (30%), B for 6 dogs (15%), F for 5 dogs (12.5%) and C for 1 dog (2.5%).

The magnitude of the patellar height decrease (ISIcls-ISIpo) was similar in each position: position E, 0.22 (0.06–0.48); position D, 0.22 (0.05–0.53); position B, 0.3 (0.11–0.6); and position F, 0.3 (0.25–0.31).

There were no differences among positions D, B, and F in PLTtc grade 4 (0–5), but there was a difference at position E (*P =* 0.292) (grade 2.5, (0–5)).

The TLAS at positions E and D did not differ (E, 0.21° (0.8–1.4°); D, 0.24° (0.82–1.44°)), but it was significantly increased in positions B and F (*P =* 0.080) (B, 0.63° (0.31–1.63°); F, 0.5° (0.38–1°)).

## Discussion

The results of our study support our first hypothesis that a systematic decrease in patellar height occurs following TPLO and AMA-based CCWO even when these procedures are performed by surgeons who are experienced in the relevant techniques. For both procedures, postoperative patellar height was found to be distributed into either the normal height class or the low height class with no TPB or PPB, but the two procedures had different effects on the patellar ligament at the two-month follow-up, with patellar height decrease and PLT occurring regardless of the osteotomy position in the TPLO group.

Assessment of decreases in patellar height following TPS alteration procedures depends on which index is most appropriate for quantifying a change [16]. During CCWO, the tibial crest moves distally, but the patellar ligament length (PLL) and the patellar length (PL) remain unchanged, resulting in similar pre- and postoperative ISIs; however, the distance between the distal pole of the patella and the tibial plateau (TP) decreases, and the GVI has been shown to be reliable and accurate for calculating changes in patellar height after this procedure and for predicting subsequent PPB [16, 21]. Following TPLO, the relationship between the patella and the TP is unchanged; thus, the GVI remains within the normal range, whereas the ISI, which reflects PLL, has been shown in previous studies to be a suitable index for assessing TPB [14, 16].

The aim of this study was not to statistically compare the magnitude of patellar height between groups because the indices used in the two groups were different but to quantify the changes in patellar height in the two groups to provide objective data that can be used by TPLO surgeons and evidence that a decrease in the height of the patella also occurs following TPLO.

PPB and TPB are well-known undesirable results following high tibial osteotomy (HTO) in human patients, and they have been correlated with poor functional outcome [14, 15]. It has been stated that in dogs that undergo CCWO, the decreased height of the patella may lead to PB [11, 13], and this is thought to be avoided with TPLO [11, 16].

PB could be a result of tibial shortening following wedge reduction with standard CCWO and has been reported to be a critical disadvantage of CCWO compared to TPLO [11–13]. Such shortening could increase the strain on the patellar ligament and increase the likelihood of secondary inflammation, but these are theoretical suggestions that have not been proven or documented in practice [6, 7, 9, 10]. In our study, precise planning of the osteotomy allowed us to superimpose the AA and MA axes postoperatively, thus reducing the median TLAS to 0°; this resulted in 82.5% of the dogs having strictly aligned axes with consecutive, predictable postoperative TPAs. Furthermore, the reduced angle of correction compared to previous recommendations for standard CCWO [4, 9, 10, 12, 23] and the positioning of the tip of the wedge at the caudodistal insertion of the medial collateral ligament rather than at the caudal cortex of the tibia, as described in previous studies [6–9], reduced the size of the wedge and limited lowering of the patella. As a result of the reduced size and precise positioning of the wedge, the median tibial shortening, even in this cohort of large dogs with large TPAs, was 3.5 mm, comparable to the mean difference in tibial length of 2.5–3 mm for CCWO documented in previous publications [6–9, 16].

In the present study, neither PLT nor patellar tendonitis (PLT = 0) was recorded at the two-month follow-up after CCWO, confirming what has been reported in previous studies concerning stifles subjected to AMA-based CCWO [7, 16]. In contrast, PLT and patellar tendonitis have been reported to occur in 80–100% of patients after TPLO [19, 20], although the position of the tibial crest remains unchanged. In our study, only 40% of the dogs in the TPLO group had PLT grade 0–2 at the two-month follow-up, with 40% having a grade higher than 4.

It has previously been suggested that PLT following TPLO procedures could be a consequence of arthrotomy and abnormal stresses on the patellar ligament following rotation of the proximal tibial segment [19, 20] and that a TPA of less than 6° may contribute to increased PLT, but this was not recorded in our case series; regardless of group, dogs with TPA > 30° had almost the same amount of rotation, the same TPA postoperatively and the same mini-arthrotomy, but they had different PLT grades at 2 months postoperatively [20].

Kowaleski et al. [18] postulated that in the TPLO procedure, the osteotomy must be on the long axis of the tibia and centred on the point that divides the intercondylar tubercles (Position E) to avoid TLAS and a subsequent patellar height decrease. The present study shows that regardless of the location in which the radial osteotomy was performed, even if it was in the correct position, a similar decrease in patellar height occurred. The patellar height decrease following TPLO is thus a consequence of the radial osteotomy followed by caudal rotation of the proximal segment of the tibia and not a result of misplacement of the osteotomy. However, the decrease in patellar height is exacerbated by osteotomy in the caudodistal position. In this study, we found that dogs with grades > 4 had greater magnitudes of patellar height decrease and TLAS.

The consequence of a decrease in postoperative ISI with TPLO is a decrease in the distance between the distal pole of the patella and the tibial crest. Thus, postoperatively, the patellar ligament and collateral ligaments are under less tension, as reported in a recent study [24], and with active quadriceps muscular contraction, fibre tearing in the patellar ligament and its paratendon could occur due to abrupt loading; this could explain the higher frequency of PLT at the level of the tibial crest observed in this study, and it confirms what has been reported previously [19, 20].

Our study also confirmed our second hypothesis, which proposes that the craniocaudal position of the patella following TPLO is different from that in a normal stifle and that the patella shifts from caudal to the AA to cranial to the AA with a reduced AMA angle with AMA-based CCWO, a position more closely related to the position found in the control animals.

As postulated by Mazdarini et al. [25], despite the fact that an increased magnitude of the AMA angle has been associated with an increased risk of CCLR based on comparisons of dogs at low risk of developing CCL disease with dogs predisposed to CCLR and on comparisons of predisposed dogs with and without CCLR [21, 22], there are insufficient data to indicate that decreasing the AMA angle by itself is an aid to joint stability [25]. However, a similar approach to AMA-based CCWO has been described recently. CORA-based levelling osteotomy (CBLO) achieves a similar rotation of the entire proximal tibial metaphysis relative to the AA, reducing the TPA by the same ratio as does AMA-based CCWO (65% of the TPA), which is less than the desired rotation needed to obtain joint stability with the TPLO procedure [26]; this induces alignment of the proximal and distal anatomic axes of the tibia and thus reduces the AMA angle. Good to excellent outcomes after CBLO, even in dogs with TPA > 30°, have been recorded in several recent studies [27–30]. Accordingly, it could be argued that although the desired planned postoperative TPA is higher with CBLO and AMA-based CCWO than with TPLO, alignment of these two axes during the change in TPA limits the secondary translation of the tibia and the caudal tibial thrust during weight bearing and thereby contributes to the stability of the stifle joint [7, 16, 22, 27, 28, 30].

The AMA angle should be taken into consideration when a TPS alteration procedure must be chosen, especially since the AMA angle and TPA are strongly correlated with TPA > 30°, with almost 80% of dogs having AMA angles > 3° [21, 22].

In this subset of dogs, following TPLO, the malalignment between the AA and the MA persists postoperatively, and the AMA angle remains greater than 3°, resulting in increased caudal displacement of the weight-bearing axis that has been recognized to cause a focal increase in joint forces at the caudal aspect of the tibial plateau, with resulting loss of compliance of supporting structures such as the joint capsule, leading to cartilage erosion [27, 31, 32].

Furthermore, as reported by Kim et al. [31], the clinical consequences of the persistence of these abnormal joint contact mechanics could explain the progression of osteoarthritis that is frequently observed in stifles treated with TPLO, particularly those with a greater magnitude of TPA [31, 33–35].

In contrast, as described for the CBLO, CCWO allows modification of the TPA while the AMA angle is reduced, resulting in alignment of the proximal tibial epiphysis on the tibial shaft with the weight-bearing forces that are transmitted through the tibial diaphysis, a situation recorded in unaffected dogs of the control group. Therefore, there is no secondary translation or “balcony” effect, as seen in dogs with TPA > 30° treated with TPLO [27–29].

CCWO should therefore be considered as a treatment for CCL-deficient canine stifles with TPA > 30° and AMA angle > 3° and may be more suitable than TPLO in this subset of dogs.

Additional studies that assess long-term clinical outcomes in dogs with CCL injury with TPA > 30° treated with either TPLO or AMA-based CCWO are warranted, as are long-term radiographic studies to determine whether osteoarthritis progresses more severely in one group and to assess a possible relationship to the magnitude of patellar height decrease, PLT and the postoperative persistence of an AMA angle > 3°.

## Conclusions

The findings of the current study support the hypothesis that TPLO and AMA-based CCWO are associated with significant patellar height decreases and no postoperative PB; however, the effects of the two procedures on PLT at 2 months differed, with patellar height decrease and PLT occurring regardless of the osteotomy position in the TPLO group. Compared to TPLO, CCWO results in reduced postoperative AMA angles and craniocaudal patellar positions that more closely resemble those of unaffected dogs, suggesting that the CCWO procedure allows us to better correct the caudal bowing of the proximal tibia that is often associated with deficient stifles in large dogs with TPA > 30°.

## Methods

### Data collection

The medical records of dogs that had been treated for CCLR in two veterinary clinics were reviewed until 40 consecutive cases of large dogs with TPA > 30° with complete sets of medical records and radiographs of the contralateral side pre- and postoperatively and at the two-month follow-up were obtained.

Two surgeons (AV and LV) performed TPLO at the Vezzoni veterinary clinic, and one surgeon (LG) performed AMA-based CCWO at the Le Vernet veterinary clinic. All three of the surgeons had completed more than 1000 procedures with the chosen technique.

Dogs eligible for inclusion weighing more than 20 kg had naturally occurring, surgically confirmed, unilateral partial or complete CCLR and no evidence of any other concurrent stifle pathology upon physical and radiographic examination.

Tibial measurements were obtained radiographically preoperatively and postoperatively on the affected side (CCLR-postop) and on the healthy contralateral side (CCLR-cls).

The proximodistal and craniocaudal positions of the patella relative to the AA were measured postoperatively on the affected side (CCLR-postop) and on the healthy contralateral side (CCLR-cls).

Finally, PLT was assessed at the two-month follow-up.

The CCLR group was compared to a control group in which the same measurements were performed.

The dogs in the control group (*n* = 40 healthy Labrador Retrievers (LR) aged > 11 years, 80 normal stifle radiographic images) were used as a control group in a previous study [21]. Both stifles were assessed, but the data for the control group were from only one side.

In accordance with a previous study [21], the GVI was divided into 4 classes (> 1.8 = patella alta, 1.25–1.8 = normal height, 0.9–1.25 = low height, and < 0.9 = PB).

Based on the mean +/− SD values of the ISI in dogs with and without CCLR, which have been previously reported, ISI < 1.9 represents low height, and ISI < 1.6 indicates PB [36, 37].

### Radiographic measurements of the tibia

**Fig. 1:**
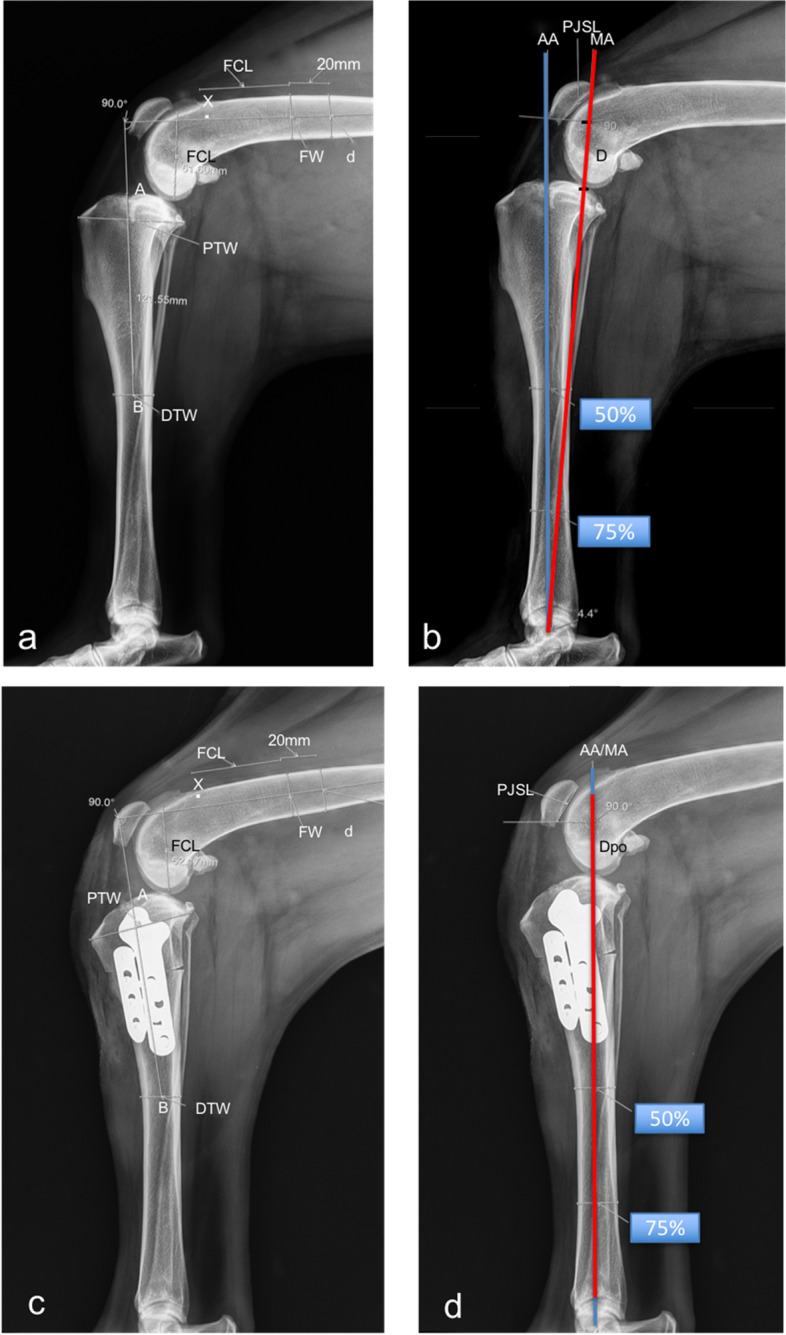
Measurements made in the AMA-based CCWO group. Mediolateral radiographs of the contralateral stifle (**a**, **b**) and postoperative mediolateral radiographs (**c**, **d**) illustrating the measurements made in the AMA-based CCWO group. **a** Measurement with the stifle joint angle at 90° according to Mostafa et al. [36] allows determination of the GVIcls (D/PJSL) and the craniocaudal position of the patella relative to the AA (**b**), and measurement of the AMA-based CCWO stifle postoperatively with the stifle joint angle at 90° (**c**) allows the calculation of the GVIpo (Dpo/PJSL). The GVIcls-GVIpo reflects a decrease in the distance between the distal pole of the patella and the tibial plateau. GVI, Guenego-Verwaerde index; cls, contralateral side; po, postoperative; A, cranial extent of the medial tibial plateau; PTW, proximal tibial width; AB = 2xPTW; DTW, distal tibial width; FCL, femoral condylar length; FW, femoral width; AA, anatomical axis; MA, mechanical axis; D, distance between the point at the intersection of the MA and the tibial plateau and the line perpendicular to the distal aspect of the patellar joint surface length (PJSL) according to Guenego et al. [16, 21]

The stifle joints of all dogs in the control group and the AMA-based CCWO group (the contralateral and postoperative stifles) were radiographed with the stifle in 90° of flexion using a previously reported method (Fig. 1a and c) [21, 36].

Briefly, the stifle joint angle was defined by the angle formed by the proximal tibial axis and the distal femoral axis according to Mostafa et al. [36] Because other tibial measurements have previously been reported to be independent of the stifle angle and the ISI has been shown to be constant over a stifle angle range of 70–110°, the stifle flexion angle was not necessarily at 90° for dogs in the TPLO group, but it was at 70–110° for the evaluation of patellar height [36].

The AMA angle (Fig. 1b) and TPA were evaluated using the methods described and used in previous reports [3, 21, 22, 38].

In the AMA-based CCWO group, the cranial wedge osteotomy (CWO) angle, the length of the cortical bone opposite the wedge, and the decrease in tibial length were recorded.

### Evaluation of patellar positions

#### Proximodistal patellar position

**Fig. 2:**
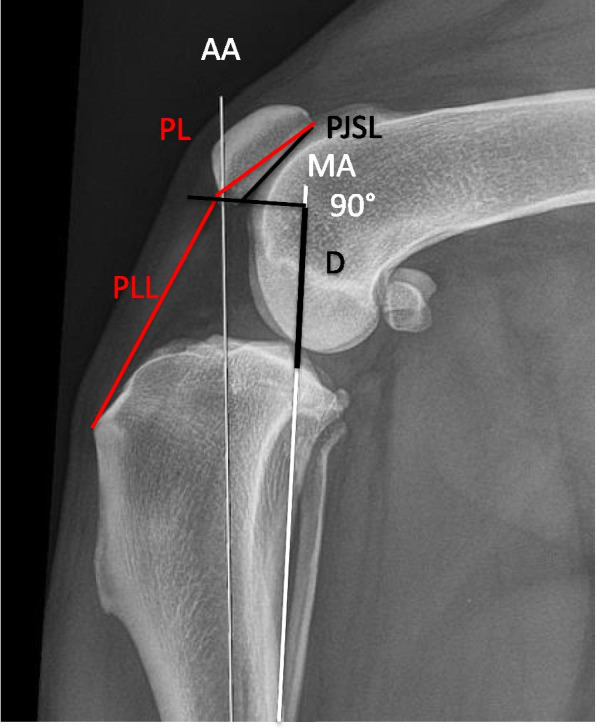
Measurements made to calculate the Insall-Salvati index (ISI) and the Guenego-Verwaerde index (GVI). Mediolateral radiograph of a normal contralateral stifle illustrating the measurements made to calculate the Insall-Salvati index (ISI) and the Guenego-Verwaerde index (GVI). The ISI is the ratio of the patellar ligament length (PLL) to the patellar length (PL), and the GVI is the ratio of D to the patellar joint surface length (PJSL). D, Distance between the point at the intersection of the MA and the tibial plateau and the line perpendicular to the distal aspect of the PJSL

Proximodistal patellar position was assessed using the ISI [15, 36, 37] and the GVI [16, 21] as previously described (Fig. 2).

Proximodistal patellar position following TPLO and AMA-based CCWO was assessed used the index most appropriate for quantifying the change associated with each procedure [16]. Because these procedures alter the relationship between the patella and the tibial crest and the TPS in different ways, ISI [15, 36, 37] is most suitable for evaluating changes that occur after TPLO, and GVI [16, 21] is most suitable for evaluating changes that occur after AMA-based CCWO, as previously described (Fig. 2) [16].

The ISI was calculated by dividing the PLL by the PL (Fig. 2), and the GVI was calculated as the ratio between the distance from the point intersecting the TP and the MA and the point at the intersection of a line drawn perpendicular to the MA and the distal point of the patellar articular surface (D) to the patellar joint surface length (PJSL) (Fig. 2).

**Fig. 3:**
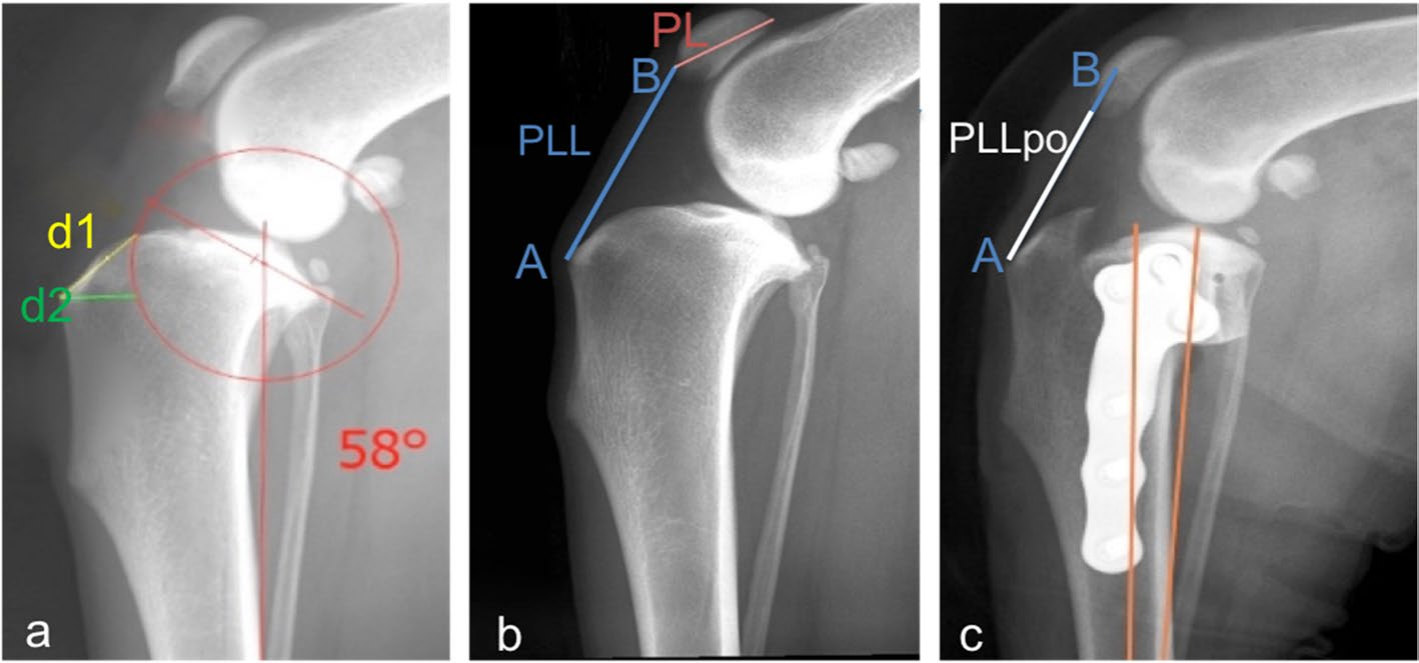
Measurements made in the TPLO group. Mediolateral radiographs of a dog in the TPLO group illustrating the measurements made during preoperative radiographic planning (d1-d2 technique) (**a**) and to determine ISIcls, where ISIcls = PLL (AB)/PL (**b**), and ISIpo, where ISIpo = PLLpo/PL (**c**). The ISIcls – ISIpo reflects a decrease in the distance between the distal pole of the patella and the tibial crest. The distance AB in Figure b is shown in Figure c to better display the patellar height decrease. ISI, Insall-Salvati index; cls, contralateral side; po, postoperative; PLL, patellar ligament length; PL, patellar length

In the TPLO group, the ISI values obtained on the contralateral side (ISIcls) (Fig. 3b) were compared to the postoperative ISI values (ISIpo) (Fig. 3c), and the magnitude of the decrease in patellar height was defined as ISIcls – ISIpo.

In the CCWO group, the GVI values obtained on the contralateral side (Fig. 1b) (GVIcls) were compared to the postoperative GVI values (GVIpo) (Fig. 1d), and the magnitude of patellar height decrease was defined as GVIcls – GVIpo.

#### Craniocaudal patellar position

The craniocaudal position of the patella was defined by the position of the patella relative to the AA and the magnitude of the AMA angle according to the data recorded in a study by Guenego et al. [21] Indeed, in the current study, in all dogs with an AMA angle above 3° the patella was caudal to the AA, and in all dogs with an AMA angle below 1.5° the patella was cranial to the AA. Based on these data, in the TPLO group, patellae were classified as caudal to the AA if the AMA angle was > 3° and as cranial to the AA if the AMA angle was < 1.5°.

### Patellar tendon thickening

PLT was evaluated at the 8-week radiographic follow-up and was defined as thickening of the patellar tendon and its paratendon.

PLT was graded on a 6-point scoring system using modifications of a previously described scoring system (0 = normal, 1 ≤ 2 times normal thickness, 2 = 2–3 times normal thickness, 3 = 3–4 times normal thickness, 4 = 4–5 times normal thickness, 5 > 5 times normal thickness) [19, 20].

**Fig. 4:**
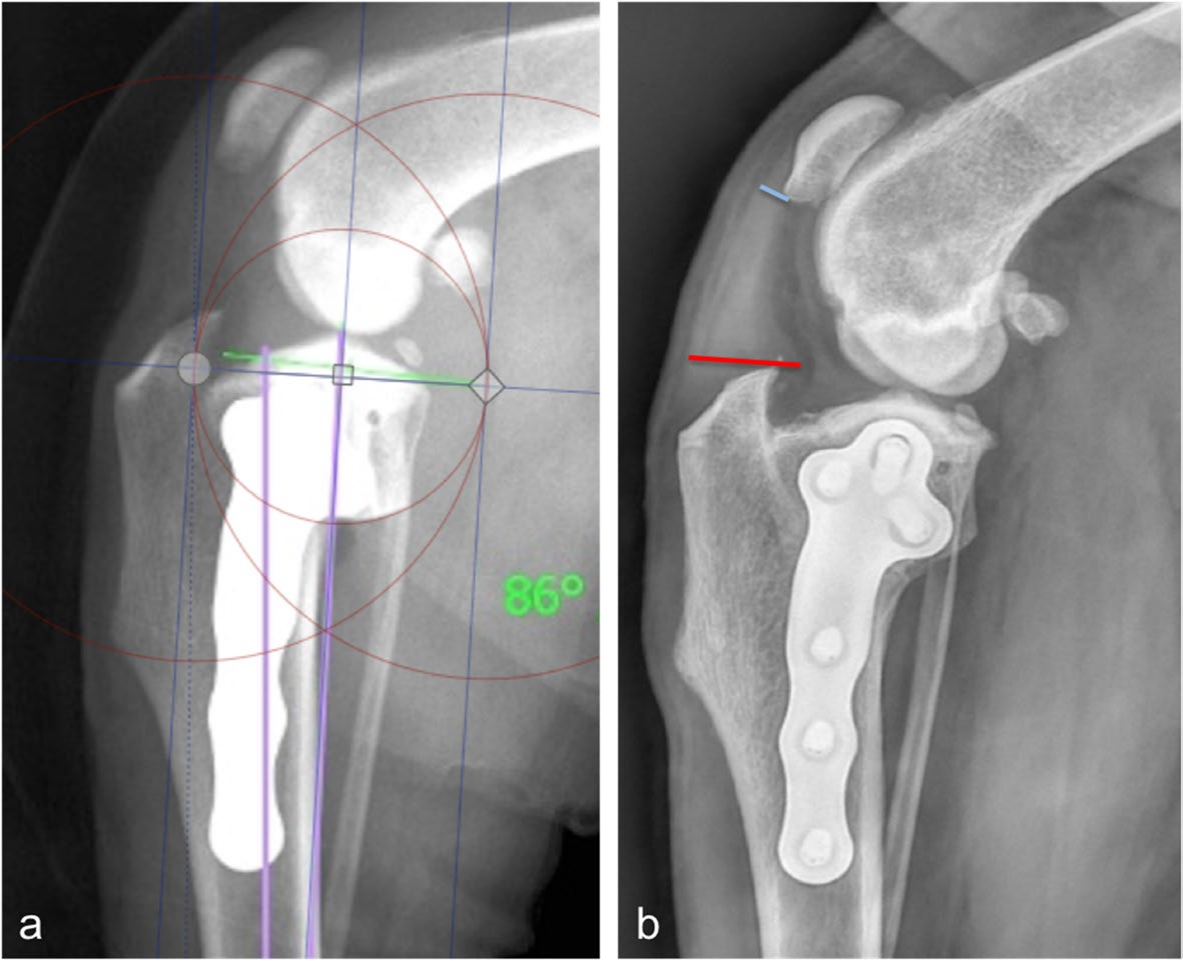
Postoperative osteotomy position and PLT in the TPLO group. Mediolateral radiographs of a stifle of a dog in the TPLO group illustrating the postoperative osteotomy position as evaluated using Pixelstick software (circles) with the centre of the osteotomy placed in position E (on the tibial long axis) (**a**) and the same stifle 2 months postoperatively with grade 5 PLT at the level of the tibial crest (red line) and grade 2 PLT at the level of the distal pole of the patella (blue line) (**b**). PLT, patellar ligament thickening

PLT was assessed at the level of the tibial crest (PLTtc) and at the level of the distal pole of the patella (PLTp) (Fig. 4b).

Dogs with PLT grades of 0 to 2, 2 to 4 and greater than 4 were pooled to identify any differences related to age, weight, sex, TPA, AMA angle, ISIpo, tibial long axis shift (TLAS), and patellar height decrease.

At 2 months postoperatively, the ISI (ISIpo2m) of the TPLO group was measured and compared to the ISI of the control group.

### Surgical procedures

For both techniques, the same standard medial approach was used, and a similar craniomedial parapatellar mini-arthrotomy was performed to allow the surgeon to inspect the cruciate ligaments and to treat the meniscal lesions according to the surgeon’s preference.

### TPLO procedure

All TPLOs were performed using a previously described technique; to maintain a consistent osteotomy position during surgery, the saw blade was centred as close as possible to the centre of the intercondylar eminence (Fig. 3a) [39]. All TPLOs were performed using an alignment jig and a cranially positioned K-wire placed adjacent to the patellar tendon to temporarily stabilize plateau rotation; the wire was removed after plate fixation [3, 18, 39].

The exact postoperative osteotomy position following TPLO was evaluated using Pixelstick software (PixelStick 2.16.0, Plum Amazing Software, LLC, Princeville, HI, USA) and a modification of a classification previously described by Kowaleski et al. [18] (Fig. 4a); the centre of the osteotomy was placed (A) on the tibial plateau slope cranial to the point dividing the intercondylar tubercles; (B) on the tibial plateau slope caudal to the point dividing the intercondylar tubercles; (C) on the tibial long axis proximal to the point dividing the intercondylar tubercles; (D) on the tibial long axis distal to the point dividing the intercondylar tubercles; (E) on the tibial long axis centred on the point dividing the intercondylar tubercles; and (F) caudodistal to the point dividing the intercondylar tubercles [18].

The differences between the TPAd and the TPApo and the TLAS, measured as the difference between the preoperative and postoperative AMA angles (AMA angle – AMA anglepo), were recorded.

Finally, the relationships among osteotomy position, the magnitude of the patellar height decrease, TLAS and PLT were evaluated.

### AMA-based CCWO procedure

**Fig. 5:**
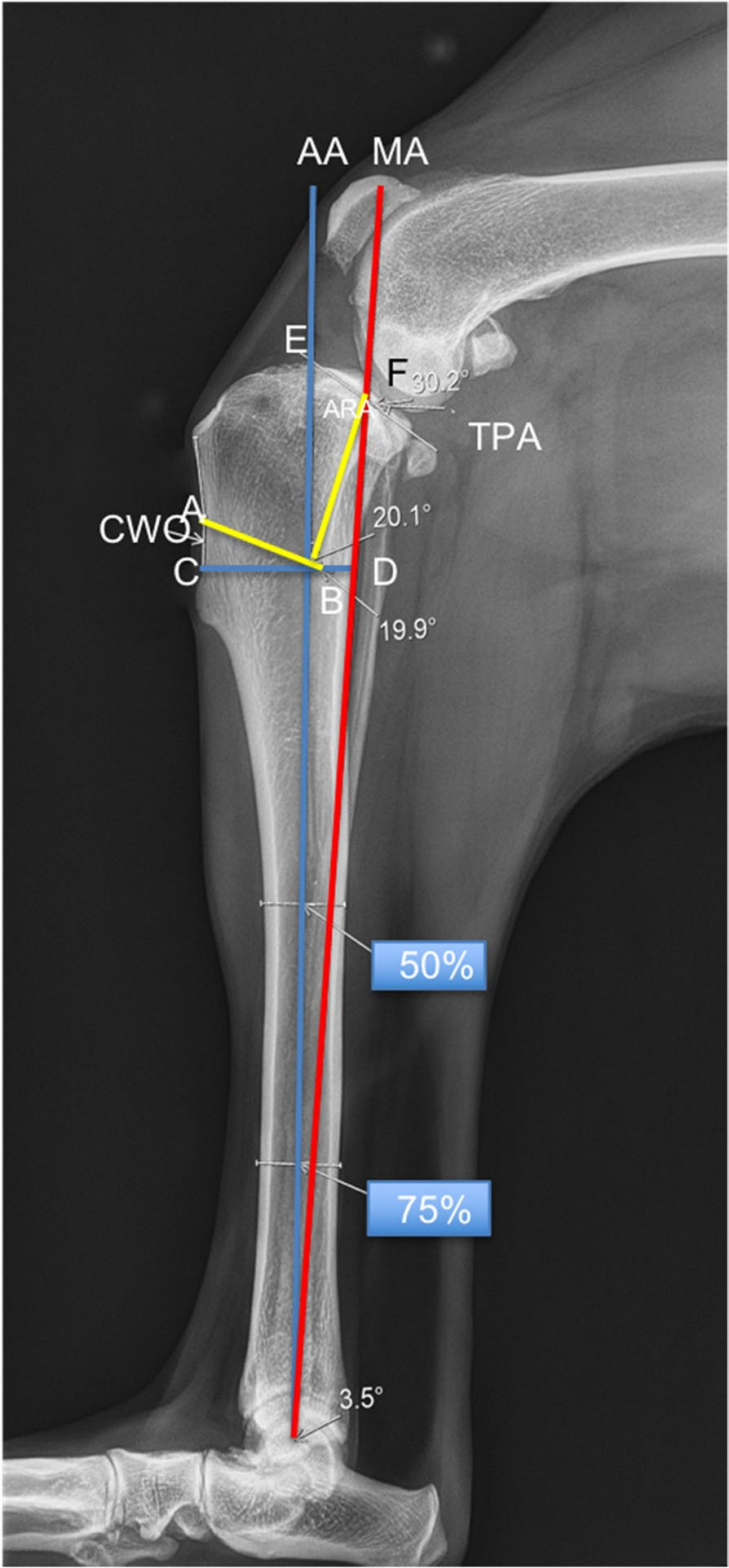
Preoperative mediolateral radiographs of a canine stifle with CCLR to illustrate AMA-based CCWO planning. AA, anatomic axis; MA, mechanical axis; AMA angle, angle between the AA and the MA. The angle EBF and the angle of rotation angulation between the AA and the MA corresponded to the planned cranial wedge osteotomy (CWO) angle (ABC angle) for reducing the tibial plateau angle (TPA). The distal osteotomy line of the CWO (CD) was perpendicular to the AA, and the proximal line (AB) was perpendicular to the BF. B is located at the caudal insertion of the medial collateral ligament. AC defines the cranial cortical length of the wedge

AMA-based CCWO has been described recently [7, 16]; it involves accurately placing the cranial wedge such that the AA and MA are superimposed after osteotomy completion, while the TPS is decreased and the TPAd is obtained (Fig. 5).

The CWO angle was defined by the magnitude of the angle (CWO = TPAx0.65) and is equal to the angle of rotation angulation (ARA) between the AA and the MA [7, 16].

TPAd was determined based on preoperative TPA as follows: TPAd = TPA – 0.65xTPA.

Elimination of 65% of the TPA was reported in two previous studies that reported 92–95% good to excellent outcomes after CCWO at 2 months recheck [4, 40]; 65% was also reported to be the magnitude of TPA correction obtained using a similar surgical TPS alteration procedure, the CBLO, which involves rotation of the entire proximal tibial metaphysis relative to the AA, thereby reducing the TPA and AMA angle and inducing alignment of the proximal and distal anatomical axes of the tibia [27, 28].

As described by Raske et al. [27] and Hulse et al. [32], elimination of 65% of the slope would retain a degree of normal cranial translation and thereby maintain the compliance of cranial soft tissues (joint capsule and fat pad) and possibly prevent the development of abrasive articular cartilage lesions seen with rotation to 6°.

All of the dogs in the CCWO group were operated on using the same procedure (AMA-based CCWO) with the same double-plating fixation technique previously described (Fig. 1d) [7, 16].

The difference between TPAd and TPApo was assessed, as was the magnitude of the TLAS, indicated by AMA angles that became negative when the AA shifted caudally to the MA.

### Statistical analyses

All statistical analyses were performed on data obtained from one limb of each dog in the control group; the results of the analysis were verified using data from both sides to accommodate the lack of independence of the samples [41].

The normality of distributions was evaluated using the Shapiro-Wilk test. For consistency, all data are presented as the median (range). Statistical analysis was performed using a nonparametric approach. Statistical comparisons across groups were made using the chi-square test, the bilateral Mann-Whitney rank test or the Kruskal-Wallis test, as appropriate.

Intra- and interobserver analyses of the AMA angle, TPA, ISI, and GVI have been performed and reported previously [21, 22].

All of the radiographs used in the present study were reviewed by a single author (X).

All statistical analyses were performed using PASW18 software (SPSS, Chicago, IL, USA).

Significance for all statistical tests was set at *P* < 0.05.

## Data Availability

The datasets used and/or analysed in the current study are available from the corresponding author on reasonable request.

## Abbreviations

CCLR: Cranial cruciate ligament rupture
TPLO: Tibial plateau leveling osteotomy
CCWO: Cranial closing wedge osteotomy
AA: Anatomical axis
MA: Mechanical axis
AMA angle: Angle between the AA and MA
TP: Tibial plateau
TPA: Tibial plateau angle
TPS: Tibial plateau slope
FCL: Femoral condylar length
FW: Femoral width
DTW: Distal tibial width
PTW: Proximal tibial width
ISI: Insall-Salvati index
PLL: Patellar ligament length
PL: Patellar length
GVI: Guenego-Verwaerde index
PJSL: Patellar joint surface length
D: Distance between the point at the intersection of the MA and the tibial plateau and the line perpendicular to the distal aspect of the PJSL
PB: Patella baja
TPB: True patella baja
PPB: Pseudo-patella baja
TLAS: Tibial long axis shift
PLT: Patella ligament thickening
